# The diagnostic role of 2-[^18^F]FDG PET/CT in critically ill patients with suspected infection of unknown origin: a real-world experience

**DOI:** 10.1007/s11547-025-02005-y

**Published:** 2025-04-21

**Authors:** Domenico Albano, Carlo Rodella, Anna Calabrò, Andor W. J. M. Glaudemans, Giorgio Treglia, Francesco Bertagna

**Affiliations:** 1 University of Brescia, Brescia, Italy; 2 Nuclear Medicine, ASST Spedali Civili Brescia, P.le Spedali Civili 1, 25123 Brescia, Italy; 3 Health Physics Department, ASST Spedali Civili, 25123 Brescia, Italy; 4 Nuclear Medicine, CRO Aviano, Aviano, Italy; 5 Department of Nuclear Medicine and Molecular Imaging, University Medical Center Groningen, University of Groningen, Groningen, The Netherlands; 6 Division of Nuclear Medicine, Imaging Institute of Southern Switzerland, Ente Ospedaliero Cantonale, Bellinzona, Switzerland; 7 Faculty of Biomedical Sciences, Università Della Svizzera Italiana, Lugano, Switzerland; 8 Faculty of Biology and Medicine, University of Lausanne, Lausanne, Switzerland

**Keywords:** PET/CT, ICU, Critically ill, FDG, Positron emission tomography

## Abstract

**Purpose:**

2-Deoxy-2-[^18^F]-fluoro-d-glucose (2-[^18^F]FDG) positron emission tomography/computed tomography (PET/CT) is a hybrid imaging tool with an emerging role in the study of several infectious and inflammatory diseases. Its role in critically ill patients admitted to the intensive care unit (ICU) is only marginally investigated. This study aimed to investigate the diagnostic performance and the clinical impact of 2-[^18^F]FDG PET/CT in the management of critically ill patients with suspected infections of unknown origin.

**Methods:**

We retrospectively included 47 ICU patients (mean age 63.6 years, 33 males) who underwent a 2-[^18^F]FDG PET/CT to look for infection foci. We calculated the diagnostic performance of PET/CT expressed as sensitivity, specificity, positive predictive value (PPV), negative predictive value (NPV) and accuracy. As reference standard, final diagnosis based on microbiological/pathologic data or on clinical follow-up was taken. Moreover, we investigated the impact of PET/CT on clinical management and the association of PET/CT with the main clinical, epidemiological and biochemical parameters.

**Results:**

The overall 2-[^18^F]FDG PET/CT sensitivity in detecting infectious foci was 80% (95%CI 64–91%), specificity 75% (95%CI 35–97%), PPV 94% (95%CI 83–93%), NPV 43% (95%CI 26–61%) and accuracy 79% (95%CI 65–90%). The absence of kidney failure, increase leukocyte count and low glucose level at the time of PET were significantly associated with true positive PET/CT. No adverse events during the transport and PET/CT procedure were registered.

**Conclusion:**

PET/CT demonstrated to be an accurate toll for the study of critically ill ICU patients with suspected infections. Kidney failure, leukocyte count and blood glucose were associated with true positive PET/CT.

## Introduction

2-Deoxy-2-[^18^F]-fluoro-d-glucose (2-[^18^F]FDG) positron emission tomography/computed tomography (PET/CT) is a hybrid imaging tool that is widely used for detection, staging, and therapy follow-up in many indications. 2-[^18^F]FDG exploits the ability to detect hypermetabolic lesions based on their increased glycolytic metabolism. Beside in oncological applications (the main field of application), 2-[^18^F]FDG PET/CT showed a significant yield also in the evaluation of inflammatory and infectious diseases [1, 2]. This imaging tool may detect the source of inflammation or infection in a timely fashion ahead of morphological changes on conventional anatomical imaging techniques, such as computed tomography (CT) and magnetic resonance imaging (MRI), map the extent and severity of disease, identify sites for tissue sampling, and assess therapy response. There is a clear diagnostic role of 2-[^18^F]FDG PET/CT in studying patients with fever and/or inflammation of unknown origin [3], bacteremia [4], vascular graft infection [5], bone and joint infections [6], endocarditis [7], etc., and new indications in infection and inflammation fields are emerging. Infections are fundamental drivers of organ failure and critical illness and the most frequent cause of sepsis in patients hospitalized at the intensive care unit (ICU) [8, 9]. These patients are by definition critically ill patients because they are in critical conditions with unstable vital signs and a higher risk of imminent death. Moreover, critically ill patients have a high risk of developing nosocomial infections [10, 11]. For these reasons, the early and accurate detection of infectious and/or inflammatory foci in these patients is crucial, with a fundamental role in guiding effective and targeted treatments. It was demonstrated that a delay in diagnosis increases the risk of death, regardless of the cause of infection [12].

A routine work-up in the ICU for the search of infections of unknown origin, especially sepsis, usually includes microbiological cultural examinations (on blood, urine and suspicious sites), blood sample biomarkers and imaging studies such as CT or ultrasound. However, in about 50% of cases, the cultures and imaging resulted negative but an infection was subsequently revealed. 2-[^18^F]FDG PET/CT is not yet routinely used in this setting despite few promising results are available in the literature [13, 14]. The limited use of PET/CT in these patients is due to the perception of the complexity of planning, preparing and transporting patients in critical conditions to perform this scan. Moreover, PET/CT is more complex than other radiological tools, like CT, related to the longer scan time, the need of a waiting time between radiotracer injection and scan, and the need of a specific preparation (such as fasting, monitoring of blood glucose level, discontinuation of certain drugs, and specific diet). Furthermore, the management of critically ill patients outside the ICU may be challenging and increases the risk of complications like hypotension crisis or O2 desaturation.

Moreover, the bio-distribution of the radiotracer is affected by several features (kidney and liver function, recent surgical procedures, presence of catheters or devices) that often are present in critically ill patients [15, 16].

This study aimed to investigate the diagnostic performance and the clinical impact of 2-[^18^F]FDG PET/CT on the management of critically ill patients with suspected infections of unknown origin.

## Methods

### Research and protocol features

This is a monocentric retrospective study approved by the local institutional ethical committee. We recruited critically ill patients in whom 2-[^18^F]FDG PET/CT scans were performed during hospitalization in the ICU between January 2021 and June 2024 using our institutional radiological information system. All patients underwent a standard diagnostic protocol including microbiological evaluation (cultures on blood, urine and other sites according to clinical suspicion), chest x-rays, imaging tools (CT and/or ultrasound and/or echocardiography) according to symptoms and clinical indications. We collected the main epidemiological (gender and age), clinical data (kidney failure, diagnosis at the time of admission in the ICU, duration of hospitalization and ICU length of stay, ongoing mechanical ventilation, blood glucose levels and duration of antibiotic therapy), biochemical data (C-reactive protein CRP, leukocyte count, LDH and cultured pathogen), imaging results and follow-up data. Moreover, we registered any adverse event that happened during transportation and scanning.

### 2-[^18^F]FDG PET/CT protocol and interpretation

All 2-[^18^F]FDG PET/CT were performed using integrated PET/CT scanners (Discovery 690 or a DST (General Electric Company—GE®—Milwaukee, WI, USA). The PET protocol followed the guidelines of the European Association of Nuclear Medicine (EANM) [17].

An activity of 3.5–4.5 MBq/Kg of radiotracer was injected intravenously followed by an uptake time of 60 min. Patients had to fast for a minimum of 6 h before the radiotracer injection, and blood glucose levels were lower than 150 mg/dL before injection, with only one case of a patient with 160 mg/dL as level. 2-[^18^F]FDG PET/CT scans were acquired from vertex to the mid-thigh with 3 min for bed position. Additional extended (including legs) scans were executed in case of suspected of disease involvement. High fat, low-carbohydrate diet 48 h before PET was done in patients with cardiac devices.

Low-dose CT was performed for attenuation correction and anatomic mapping with 120 kV and 80 mAs as parameters. The reconstructions were performed in a 256 × 256 matrix and 60 cm field of view. For D-690, time-of-flight (TOF) and point spread function (PSF) were used as reconstruction algorithms; filter cutoff 5 mm, 18 subsets; and 3 iterations. For D-STE, ordered subset expectation maximization (OSEM) was applied; filter cutoff 5 mm; 21 subsets; and 2 iterations.

2-[^18^F]FDG PET/CT scans were interpreted by a nuclear medicine physician (DA) with experience in inflammation and infection imaging. For PET interpretation, every focal radiotracer uptake dissimilar from physiological distribution and background was considered as pathological/positive. As reference organs, we considered mediastinal blood pool and liver. Uptake higher than these two references was considered as suspected for inflammatory/infectious disease.

### Reference standard

The final clinical diagnosis, which was used as the reference standard, was made retrospectively, considering all available clinical information. This information included histological data if available, clinical course, laboratory, microbiology, imaging findings and follow-up. The 2-[^18^F]FDG PET/CT results were then compared with the final clinical diagnosis and interpreted as true positive, false positive, true negative or false negative.

Scans were considered true positive when PET/CT findings were confirmed by further investigations as the final diagnosis; false positive when 2-[^18^F]FDG PET/CT results were not confirmed to be the cause of disease; true negative if 2-[^18^F]FDG PET/CT produced a normal scan with further investigations or clinical follow-up ruling out any disease; false negative if 2-[^18^F]FDG PET/CT did not revealed any site of increased pathological uptake, but disease was subsequently detected by other diagnostic modalities.

### Evaluation of clinical impact of 2-[^18^F]FDG PET/CT

2-[^18^F]FDG PET/CT was considered to be clinically impactful and helpful if additional lesions were detected by 2-[^18^F]FDG PET/CT which were not revealed by other examinations, and if these findings changed the management (such as to guide surgery or to start antibiotic therapy) or if the scan ruled out false positive findings detected at other examinations avoiding unnecessary therapies or invasive procedures. Alternatively, 2-[^18^F]FDG PET/CT results were considered to have no clinical impact if PET/CT and other examination reports were concordant, or a discordant finding among them did not influence the subsequent patient management.

### Statistical analysis

The software that we used for the statistical analysis was MedCalc version 19 for Windows (Ostend, Belgium). All continuous variables were checked for normal distribution using Kolmogorov–Smirnov tests. The categorical variables were represented as simple and relative frequencies, and the numeric variables were represented as mean, standard deviation (SD) and range.

Based on the final diagnosis as a reference, sensitivity (SE), specificity (SP), negative predictive value (NPV), positive predictive value (PPV), accuracy (AC), positive likelihood ratio (PLR) and negative likelihood ratio (NLR) were calculated based on Bayes’s law, with 95% confidence intervals (CIs).

The relationship between PET diagnostic performances and the main clinical/biochemical/epidemiological variables was investigated with a univariate logistic regression with 2-[^18^F]FDG PET/CT result as dependent feature and other variables as independent. For this analysis, we dichotomized true positive PET/CT and all other PET/CT findings. Corresponding odds ratios (ORs) and 95% CIs were calculated, and p value less than 0.05 was considered statistically significant. Variables with *p* value ≤ 0.10 on univariate analysis were included in the stepwise multivariate logistic regression model.

## Results

### Patients’ features

We included 47 critically ill patients: Among them, there was a higher prevalence of male (*n* = 33) and the average age was 63.6 years (range 11–89). Kidney failure was registered in seven patients. Mechanical ventilation was ongoing at the time of PET/CT in eight cases. Reasons for ICU admission were very heterogeneous ranging from septic shock as the most common cause (n = 13), followed by complications prior to surgery (*n* = 8), stroke (*n* = 5), trauma, cardiogenic shock and respiratory insufficiency (*n* = 4). Other causes are summarized in Table 1. Mean CRP, leukocyte count and LDH were 63.8 mg/L, 9.34 × 10^9/L and 105.9 mg/dL, respectively. Microbiological cultures resulted positive in 55% of cases, with Staphylococcus aureus (*n* = 8), Escherichia Coli (*n* = 5) and Klebsiella pneumoniae being the most frequent pathogens. The average duration of antibiotic therapy before PET/CT scan was 6.1 days (range 1–10). The mean hospitalization length (including ICU stay and hospitalization in other departments) was 26.5 days (range 9–62), the mean ICU stay was 14.9 days (3–39 days), and the mean time in ICU before PET/CT was 8.2 days (range 2–27 days). Mean blood glucose level was 106 mg/dL. Eight patients died during hospitalization. The main characteristics of our population are summarized in Table 1.

**Table 1:** Patients’ main features

	n (%)	Mean (range)
Age, mean		63.6 (19–89)
Gender M:F	33 (70%):14 (30%)	
Kidney failure	7 (15%)	
Post-transplantation	11 (23%)	
Days of hospitalization		26.5 (9–62)
Days in ICU before PET/CT		8.2 (2–27)
Total days in ICU		14.9 (3–39)
Admission diagnosis		
*Septic shock*	13 (28%)	
*Complications prior surgery*	8 (17%)	
*Stroke*	6 (13%)	
*Trauma*	4 (8%)	
*Cardiogenic shock*	4 (8%)	
*Respiratory insufficiency*	4 (8%)	
*Subarachnoid hemorrhage*	3 (6%)	
*FUO*	2 (4%)	
*Liver failure*	1 (2%)	
*Kidney failure*	1(2%)	
*Endocarditis*	1 (2%)	
Cultures positive	26 (55%)	
*Staphylococcus aureus*	8 (31%)	
*Escherichia Coli*	5 (19%)	
*Klebsiella pneumoniae*	4 (15%)	
*Enterococcus faecium*	3 (12%)	
*Staphylococcus constellatus*	2 (8%)	
*Staphylococcus hominis*	1 (4%)	
*Staphylococcus epidermidis*	1 (4%)	
*Staphylococcus infantarius*	1 (4%)	
*Pneumocystis jirovecii*	1 (4%)	
Duration of antibiotic therapy before PET/CT (days)		6.1 (1–10)
CRP (mg/L)		63.8 (0.6–372)
Leukocyte count (× 10^9/L)		9.34 (3.3–25.3)
LDH (U/L)		259 (131–1518)
Blood glucose level at PET/CT (mg/dL)		106 (45–160)
Mechanical ventilation	8 (17%)	
Death during hospitalization	8 (17%)	

*M* male, *F* female, *CRP* c-reactive protein, *LDH* lactate dehydrogenase, *ICU* intensive care unit, *FUO* fever of unknown origin, *n* number

### Diagnostic performances of 2-[^18^F] FDG PET/CT

Using the final diagnosis derived from all the clinical data available as reference, 32 2-[^18^F] FDG PET/CT were considered as true positive (TP), 6 as true negative (TN), 2 as false positive (FP) and 8 as false negative (FN). SE, SP, PPV, NPV, AC, PLR and NLR of 2-[^18^F] FDG PET/CT were 80% (95%CI 64–91%), 75% (95%CI 35–97%), 94% (95%CI 83–93%), 43% (95%CI 26–61%), 79% (95%CI 65–90%), 3.2 (0.95–10.27) and 0.27 (0.13–0.56) (Table 2).

**Table 2:** Diagnostic performances of 2-[^18^F]FDG PET/CT

	Value	95% CI
True positive	32	
True negative	6	
False positive	2	
False negative	8	
Sensitivity	80%	64.35-90.95%
Specificity	75%	34.91-96.81%
Positive predictive value	94%	82.67-92.52%
Negative predictive value	43%	26.4–61.06%
Accuracy	79%	65.01-89.53%
Positive likelihood ratio	3.2	0.95–10.73
Negative likelihood ratio	0.27	0.13–0.56

*CI* confidence interval

**Fig. 1:**
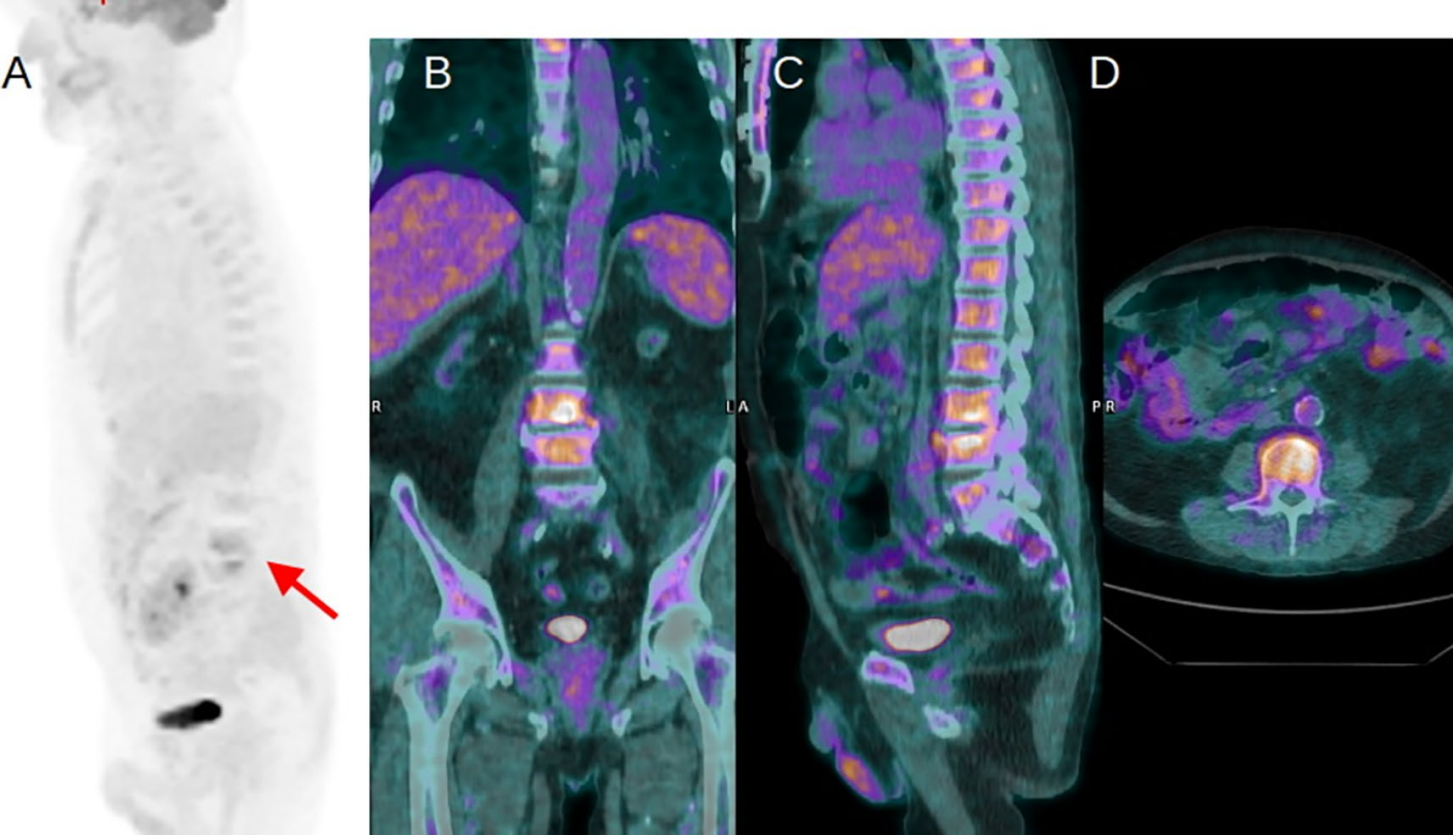
A 69-year-old man was admitted to the ICU with fever and fatigue after a prior abdominal surgical procedure. Abdominal ultrasound and thorax-abdominal CT were negative. Blood cultures were positive for *Staphylococcus aureus.* Maximum intensity projection PET (**A**) revealed the presence of an increased uptake in the column corresponding to the third and fourth lumbar vertebra. Subsequent coronal (**B**), sagittal (**C**) and axial (**D**) PET/CT fused images confirmed the increased 2-[^18^F]FDG uptake in the intervertebral disk and adjacent vertebra suspected for spondylodiscitis

In regard to true positive 2-[18F] FDG PET/CT scans, the final diagnoses were pneumonia (*n* = 9), endocarditis (*n* = 4), vascular graft infection (*n* = 4), osteomyelitis (*n* = 3), spondylodiscitis (*n* = 3), prosthetic joint infection (*n* = 3), abscesses (n = 2), autosomal dominant polycystic kidney disease (*n* = 1), pancreatitis (*n* = 1), hepatitis (*n* = 1) and diverticulitis (*n* = 1) (Fig. 1).

**Fig. 2:**
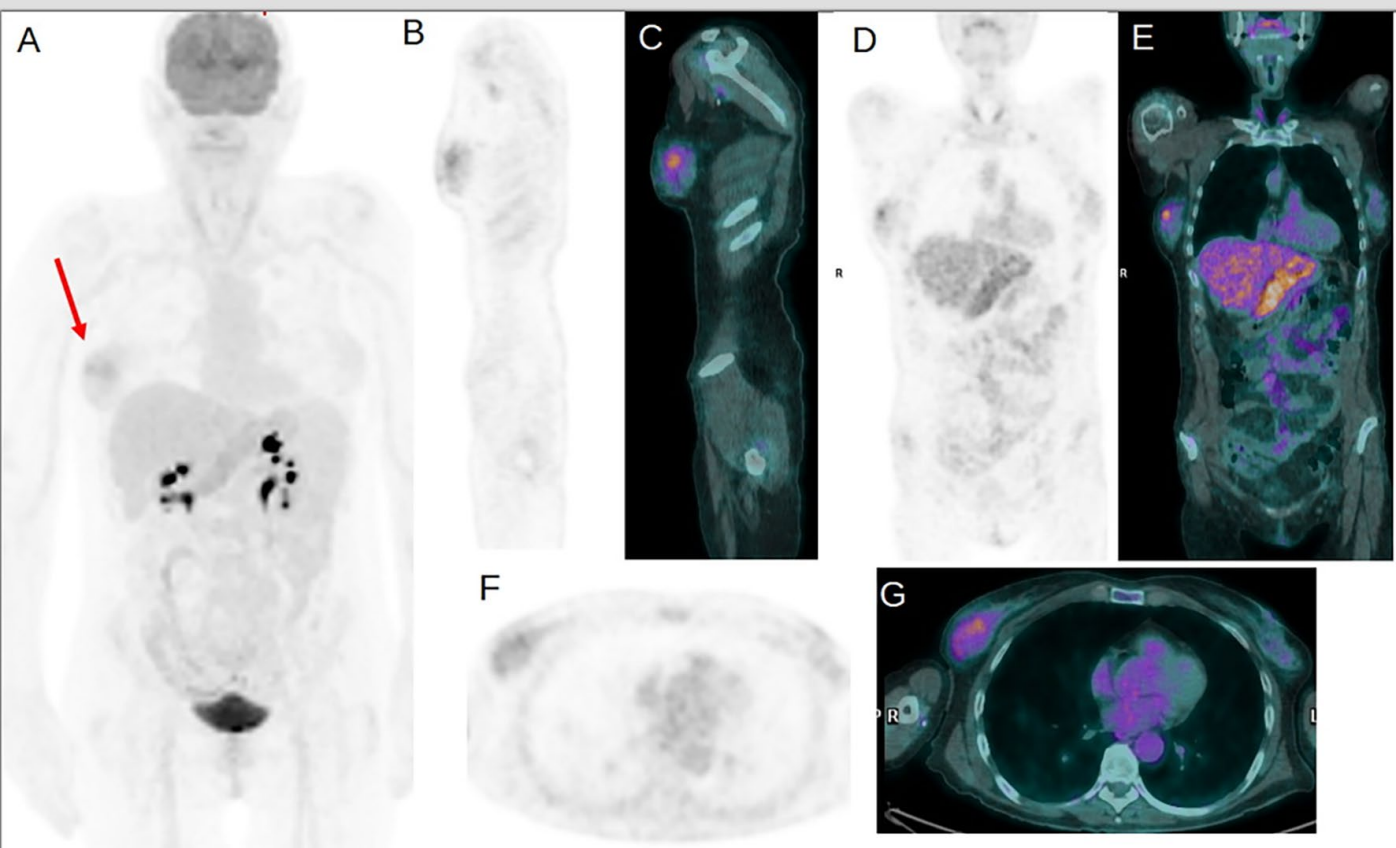
A 55-year-old woman was admitted to the hospital for subarachnoid hemorrhage and negative microbiological cultures, negative CT and echocardiography performed 2-[^18^F]FDG PET/CT for the search of any pathological uptake. Maximum intensity projection PET (**A**) revealed the presence of an increased uptake in the right breast (black arrow). Sagittal PET (**B**) and PET/CT (**C**), coronal PET (**D**) and PET/CT (**E**), axial PET (**F**) and PET/CT (**G**) confirmed that this focal uptake corresponded to a hypodense lesion with a central area of relatively reduced uptake on the right breast. This uptake was judged suspected for abscess. Subsequent breast biopsy demonstrated the benign nature of this lesion. The final diagnosis of this patient was encephalitis, but PET showed a physiological brain uptake without any area or reduced or increased uptake

In the eight patients with false negative PET/CT, the final diagnosis was pneumonia in two cases, nephritis in one case, myelitis in one case, colitis in one case, Behcet’s disease in one case, spondylodiscitis in one case, and dermo-hypodermatitis in one case. On the other hand, considering the two patients with false positive findings, in one patient, the final diagnosis was encephalitis but not recognized by 2-[^18^F] FDG PET/CT. Instead, 2-[^18^F] FDG PET/CT revealed a focal mammary uptake suspected for abscess but subsequent histological analysis revealed its benign nature (Fig. 2). In the other patient with a false positive PET/CT scan, this examination revealed a periprosthetic hip uptake not confirmed by white blood cells scintigraphy but a final diagnosis of septic thrombophlebitis was achieved.

### 2-[^18^F] FDG PET/CT clinical impact

No adverse events during transportation to and from the PET/CT department and in the PET/CT center were registered. A team consisting of an anesthesiologist physician and/or nurse anesthesist was present during the transport and stay in the nuclear medicine facilities during the uptake time and PET acquisition. 2-[^18^F]-FDG PET/CT had a positive impact in the patient’s management in 29 patients (62%), specifically in 25/32 true positive and 4/6 true negative PET/CT scans, guiding surgery in four cases (one diverticulitis, one endocarditis, one vascular graft infection and one pancreatitis) and modifying antibiotic treatment in 25 cases (starting a new antibiotic or modulating an existing antibiotic regimen in 21 patients and interrupting an antibiotic therapy in the remaining four cases) (Table 3).

**Table 3:** Summary of the impact of PET/CT on patient management

	*n* (%)
Positive impact	29 (62%)
Surgery	4 (14%)
Interruption of antibiotic therapy	4 (14%)
Modification of antibiotic therapy	21 (72%)
No impact	18 (38%)

*N* number

### Variables associated with 2-[^18^F] FDG PET/CT findings

On univariate logistic regression, the presence of kidney failure, CRP level, leukocyte count and blood glucose levels were significantly associated with PET/CT reports (Table 4). Mean blood glucose level was 114 mg/dL in patients with TN, FP and FN 2-[^18^F]-FDG PET/CT scans and 100 mg/dL in TP 2-[^18^F]-FDG PET/CT scans. CRP levels and leukocyte counts were significantly higher in patient with TP PET/CT scans than others (86.3 vs 23.7 mg/L, 10.1 × 10^9/L vs 7.65 × 10^9/L). Instead, only 2/32 (6%) patients with TP PET/CT had kidney failure compared to 5/15 (33%) of all other scans. All other parameters (age, gender, days of hospitalization, length of stay in the ICU before PET, total days of ICU, antibiotic therapy duration, LDH levels and the presence of mechanical ventilation) were not related with PET/CT results (Table 4**)**. On multivariate logistic regression analysis, kidney failure, leukocyte count and blood glucose levels confirmed to be independent variables (*p* = 0.043, *p* = 0.034 and *p* = 0.041, respectively).

**Table 4:** Logistic regression analysis

	Univariate OR (95%CI)	*p* value	Multivariate OR (95%CI)	*p* value
Age, mean	1.351 (0.600–4.650)	0.483		
Gender M:F	1.832 (0.422–7.953)	0.723		
Kidney failure	0.116 (0.019–0.702)	0.014	0.025 (0.007–0.890)	0.043
Days of hospitalization	1.201 (0.701–2.324)	0.224		
Days in ICU before PET/CT	1.333 (0.676–3.001)	0.465		
Total days in ICU	1.090 (0.786–1.801)	0.191		
Duration of antibiotic therapy before PET/CT (days)	1.700 (0.545–6.002)	0.662		
CRP (mg/L)	1.026 (1.006–1.051)	0.012	1.053 (0.998–1.118)	0.058
Leukocyte count (× 10^9/L)	1.216 (1.09–1.760)	0.003	1.482 (1.029–2.135)	0.034
LDH (U/L)	1.193 (0.810–1.345)	0.163		
Blood glucose at time of PET/CT (mg/dL)	0.877 (0.451–0.987)	0.020	0.945 (0.896–0.997)	0.041
Mechanical ventilation	1.501 (0.701–7.789)	0.546		

*M* male, *F* female, *CRP* c-reactive protein, *LDH* lactate dehydrogenase, *ICU* intensive care unit, *OR* odds ratio, *CI* confidence interval

## Discussion

In this study, we focused on the potential role of 2-[^18^F]FDG PET/CT in critically ill patients with a suspected infection, and we demonstrated a good diagnostic performance of 2-[^18^F]FDG PET/CT expressed as SE of 80%, SP of 75%, PPV of 94%, NPV 43% and ACC of 79%. Previous data regarding 2-[^18^F]FDG PET/CT diagnostic usefulness in critically ill patients with suspected infection were minimal, the populations analyzed were very small, and performances reported were very heterogeneous [18–22] (Table 5). SE was usually high, ranging from 85%-91%, while SP ranged from 50–88%. Of course, evaluating critically ill patients is challenging because of their serious health condition, the possible presence of multiple medical devices and catheters and complicated inflammatory conditions, which may interfere with the interpretation of 2-[^18^F]FDG PET. One of the advantages of 2-[^18^F]FDG is the ability to detect several different diseases, both oncological and not oncological. In this kind of patients, with many potential sources of disease, this can represent a strength. One of the limitations of 2-[^18^F]FDG PET/CT is the evaluation of infectious foci in sites of physiological tracer uptake (e.g., brain, urinary tract, skin) or areas under the resolution power of the scanners (small vessel, or in general lesion less than 5 mm of diameter). This could explain some of false negative findings reported in this study, like dermo-hypodermatitis, myelitis, small-vessel vasculitis and nephritis. This is a fundamental point to consider in these patients because not all infectious/inflammatory diseases may be accurately investigated with PET/CT. However, some of these issues might be solved with the introduction of new technology, such as “digital” or long-axial field of view tomographs. About the other cause of false negative PET/CT, we described two cases of pneumonia and one of spondylodiscitis. In cases of pneumonia, the lung alterations were characterized by ground-glass opacities that for definition are not FDG-avid. About spondylodiscitis, the reason of absence of uptake is not perfectly clear because usually this kind of disease presents significant radiotracer uptake.

**Table 5:** Summary of studies in the literature on the diagnostic performances of FDG PET/CT in critically ill patients

Author	N°	TP	TN	FN	FP	Sensitivity	Specificity	Accuracy	Helpfulness
Simons	35	21	11	0	3	100%	79%	91%	91%
Kluhe	18	11	4	0	3	100%	57%	83%	61%
Mandry	17	11	2	2	2	85%	50%	76%	71%
Pijl	30	20	7	2	1	91%	88%	90%	37%

Another limitation of the method is the reduced specificity in discriminating between infection and non-septic inflammation. 2-[18F]FDG PET/CT was demonstrated to be helpful in 37–91% (average 65%) of cases. These data were concordant with our findings where we revealed the clinical usefulness of PET/CT in 62% of patients. However, the definition of clinical helpfulness is quite different in each article, and this difference reduces the possibility of performing a comparison. Kluge et al. [18] and Simons et al. [20] considered only true positive PET scans as clinically useful independently from the impact of PET findings in changing patient management (such as new invasive procedures and change of antibiotic therapy). Instead Pijl et al. [21] and Mandry et al. [19] defined 2-[18F]FDG PET/CT scan as helpful only if PET findings modified patients’ treatment. In our work, we shared the definition of Pijl et al. [21] and Mandry et al. [19] because the effective helpfulness of an imaging tool should be measured if the patients’ management would be different without that examination. The most frequent impact of 2-[18F]FDG PET/CT in clinical management was the change in antibiotic therapy or the start of a new antibiotic drug, due to its usefulness in recognizing the site of infection.

To better understand the variables associated with 2-[^18^F]FDG PET/CT results, we investigated the main clinical, epidemiological and blood findings with a significant correlation at multivariate analysis with the presence of kidney failure, leukocytes count and blood glucose level at the time of PET. Only one study [21] investigated the relationship between kidney function status and 2-[^18^F]FDG PET/CT results demonstrating that kidney insufficiency causes a poor quality image and reduces the probability of having an accurate PET scan. It is well known that renal failure may reduce background clearance of radiopharmaceuticals causing a higher background activity and lower contrast between pathological lesions and background [23–25]. This different bio-distribution may potentially reduce the reader’s ability to analyze PET images. How much renal insufficiency affects background uptake of 2-[^18^F]FDG is probably related to the severity of renal insufficiency, but this point is not yet clear. Among blood samples, we focused on CRP and leukocyte count because they were reported to be often associated with diagnostic yield in recognizing infectious of inflammatory foci at PET/CT in non-ICU patients [3, 26–28]. Concerning critically ill patients, only one research [21] included CRP and leukocyte count in adults in their analysis, but no significant association was revealed. On the other hand, in a pediatric study [29], a significant correlation with CRP was demonstrated. In our analysis, both variables were significant at univariate logistic regression but only leukocyte count confirmed this significance at multivariate test. A high level of leukocyte count may be an indirect sign of infection and explain the association with the probability of detecting focal 2-[^18^F]FDG uptake at PET/CT. Plasma glucose levels are recommended to be as low as possible and in any case less than 200 mg/dL. Even though the effect of hyperglycemia on 2-[^18^F]FDG uptake may probably be less marked on inflammatory and infective cells compared to neoplastic cells [30], recent research demonstrated that the diagnostic role of 2-[^18^F]FDG PET/CT in bacteremia of unknown origin was lower in patients with moderate to severe hyperglycemia than normoglycemic ones [31]. Thus, it seems reasonable to aim at a lower blood glucose threshold in patients with suspected infectious diseases. In our analysis, no patients had glycemia levels higher than 200 mg/dL, and blood glucose levels were less than 150 mg/dL in all cases except one (160 mg/dL). Despite these optimal values, there was a significant impact of blood glucose level in 2-[^18^F]FDG PET/CT performances. The mean blood glucose level was 114 mg/dL in patients with true negative, false positive and false negative 2-[^18^F]-FDG PET/CT, while it was 100 mg/dL in true positive 2-[^18^F]-FDG PET/CT. ICU patients are usually treated with parenteral or enteral nutrition, and in case of difficult glycemic control, a continuous insulin infusion is a common practice. The risk of high glucose level at the time of PET/CT or an incorrect withdrawal of insulin is not negligible and needs to be carefully evaluated. Our findings support that 2-[^18^F]-FDG PET/CT is an accurate, practical and safe imaging tool to identify infections in ICU patients. Among our patients, no adverse event during transportation or in the nuclear medicine facilities were registered. In other studies, only a few such events are described, usually desaturation or hypotension. This rate seems to be lower than that reported in CT [32, 33], despite a direct comparison is difficult for the different sample included and tool characteristics. The technological progress in nuclear medicine with the introduction of “new total body” PET scanners could be a significant gain in this field. Conventional PET/CT systems usually have a 20-cm-wide detector ring, and the scan normally takes 25–30 min according to the patient height and time for bed position. With total body scanners, detector ring measures up to 200 cm, and a true whole-body PET/CT can be performed in a few minutes with superior image quality compared to current PET/CT systems [34, 35]. In problematic and critical patients like ICU patients, a gain in time and comfort may be a big advantage. The problem of the long uptake time of 2-[^18^F]-FDG and the need for critically ill patients to stay outside the ICU for a long period could be tackled by the administration at the ICU department. This required a good standardized operating procedure within your hospital and the need of a license to administer radioactivity at the ICU department. Potential limitations of PET/CT in the study of these patients are the need of specific preparation that sometimes requires a relatively long time, such as in patients with cardiac devices who need a low-carbohydrate diet. According to EANM guidelines, patients should fast for at least 6 h before FDG PET (including parental and enteral nutrition), and no glucose infusion is allowed during this time period. Another limitation of 2-[18F]FDG PET/CT is the impossibility of performing a scan at any time, independently from particular preparations. In many hospitals, PET/CT is only performed on business days, and not in the weekends. However, guidelines for bacteremia require FDG PET/CT imaging within 72 h, so these could still be followed.

Unfortunately, the perceived high cost of the method could influence its clinical use in critically ill patients. Interestingly, a recent cost-effectiveness study including also critically ill patients demonstrated that 2-[18F]FDG PET/CT could be cost-effective in diagnosing fever and inflammation of unknown origin [36].

This research has some limitations like the retrospective nature of the design which causes a potential bias in the selection of the patients, the relatively small number of patients recruited (despite the highest number in the literature) which limits the statistical power of our analyses, and the heterogeneous features of patients as far as epidemiological and clinical aspects. For these reasons, other investigations on larger populations are shareable.

## Conclusions

2-[^18^F] FDG PET/CT demonstrated to be an accurate and safe imaging tool for the investigation of critically ill ICU patients with suspected infections. Kidney function status, leukocyte count and blood glucose level were significantly correlated with true positive PET/CT results. Moreover, 2-[^18^F] FDG PET/CT was shown to be clinically useful in more than half of patients.

## Data Availability

The datasets generated during and/or analyzed during the current study are available from the corresponding author on reasonable request.
